# Hypoxia: molecular pathophysiological mechanisms in human diseases

**DOI:** 10.1007/s13105-022-00912-6

**Published:** 2022-07-23

**Authors:** Ylenia Della Rocca, Luigia Fonticoli, Thangavelu Soundara Rajan, Oriana Trubiani, Sergio Caputi, Francesca Diomede, Jacopo Pizzicannella, Guya Diletta Marconi

**Affiliations:** 1 Department of Innovative Technologies in Medicine & Dentistry, University “G. d’Annunzio” Chieti-Pescara, Chieti, Italy; 2 Department of Biotechnology, Karpagam Academy of Higher Education, Coimbatore, India; 3 Cardiology Intensive Care Unit, “Ss. Annunziata” Hospital, ASL02 Lanciano-Vasto-Chieti, Chieti, Italy; 4 Department of Medical, Oral and Biotechnological Sciences, University “G. d’Annunzio” Chieti-Pescara, Chieti, Italy

**Keywords:** Hypoxia, Molecular mechanisms, Biomaterials, Stem cells

## Abstract

Hypoxia, a low O_2_ tension, is a fundamental feature that occurs in physiological events as well as pathophysiological conditions, especially mentioned for its role in the mechanism of angiogenesis, glucose metabolism, and cell proliferation/survival. The hypoxic state through the activation of specific mechanisms is an aggravating circumstance commonly noticed in multiple sclerosis, cancer, heart disease, kidney disease, liver disease, lung disease, and in inflammatory bowel disease. On the other hand, hypoxia could play a key role in tissue regeneration and repair of damaged tissues, especially by acting on specific tissue stem cells, but their features may result as a disadvantage when it is concerned for neoplastic stem cells. Furthermore, hypoxia could also have a potential role in tissue engineering and regenerative medicine due to its capacity to improve the performance of biomaterials. The current review aims to highlight the hypoxic molecular mechanisms reported in different pathological conditions to provide an overview of hypoxia as a therapeutic agent in regenerative and molecular therapy.

**Graphical abstract:**

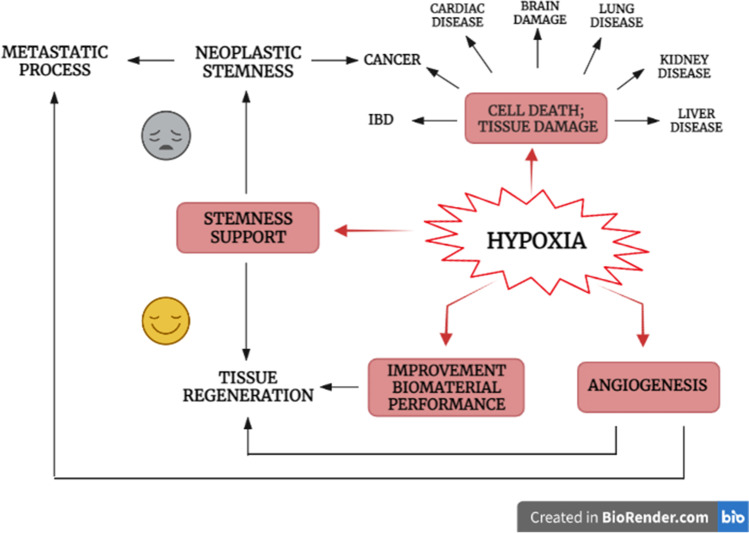

## Introduction

The term hypoxia refers to the condition where tissues are exposed to oxygen deficiency, which compromises the tissue homeostasis, an essential phenomenon for the maintenance of cellular health. This state can occur in response to a decrease in tissue oxygenation following a low blood supply due to circulatory problems and as a result of lowering hemoglobin and low blood oxygen levels. The low blood oxygen level may involve a specific area of the organism (tissue hypoxia) or it may affect the whole body (generalized hypoxia). Furthermore, hypoxia can become an acute phenomenon or it can gradually lead to chronic hypoxia. Sustained chronic hypoxia initiates a series of physiological adaptations to maintain oxygen homeostasis. Examples include increased red blood cell production (to improve oxygen-carrying capacity), the formation of new blood vessels (to facilitate the transport of oxygenated blood to tissues), and metabolic reprogramming of cells (to reduce consumption of oxygen and maintain redox homeostasis in the conditions of prolonged oxygen deprivation) [98]. In addition, 10% of individuals suffer from chronic intermittent hypoxia (IH), a condition in which the human body is temporarily deprived of an adequate supply of oxygen in the blood due to obstructive sleep apnea (OSA), a highly prevalent respiratory disorder characterized by periodic cessation of breathing during sleep [95, 101]. While many people who live at high altitudes adapt to prolonged hypoxia, those with intermittent hypoxia exhibit maladjustments that result in a variety of conditions including hypertension with a strong correlation between the frequency of apnea events and hypertension [88].

The purpose of the current review was to evaluate the involvement of hypoxia condition in various pathological conditions in order to assess the promising role hypoxia as a therapeutic in disease management.

## Hypoxia: molecular mechanism and target genes

**Fig. 1:**
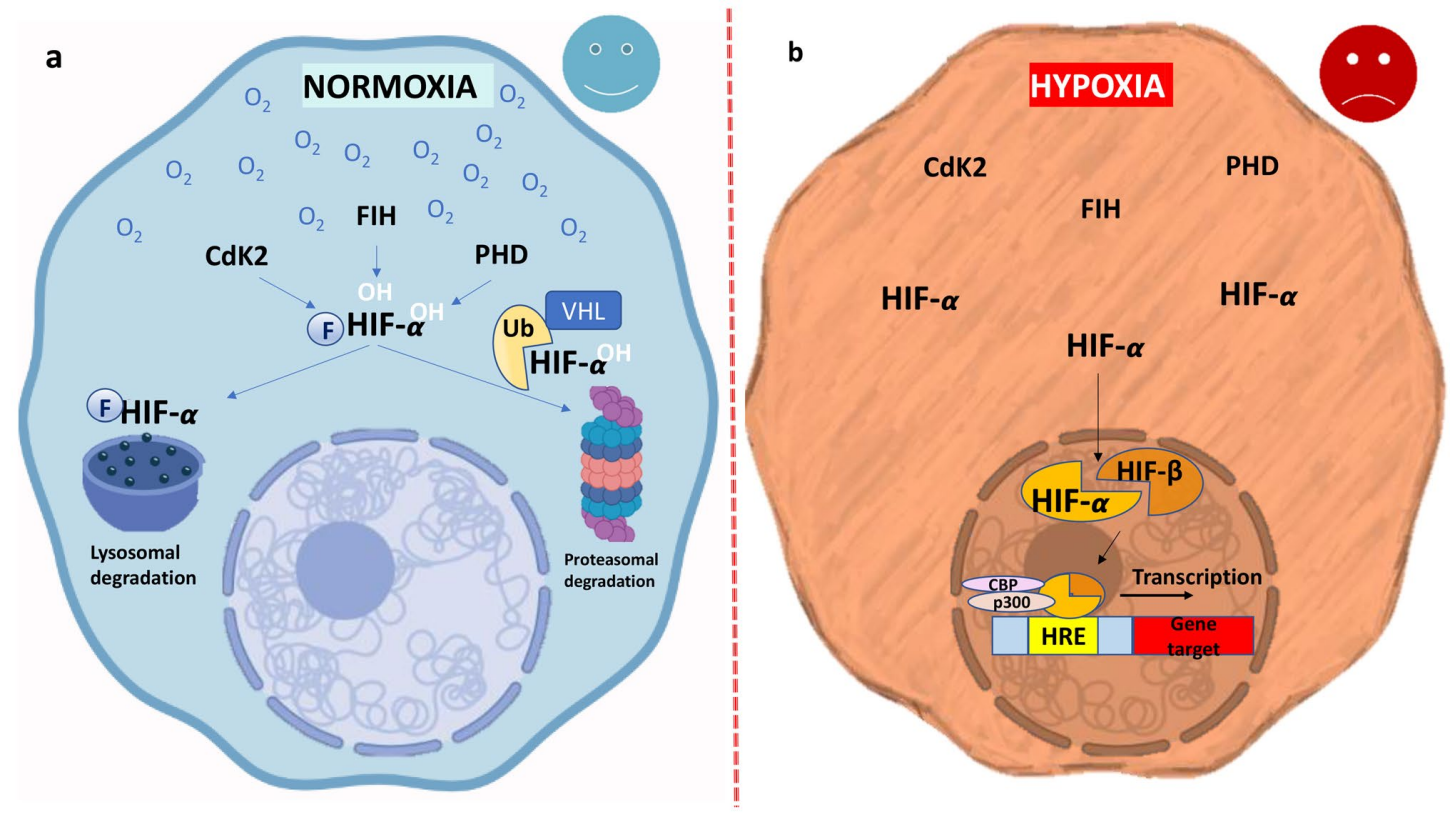
HIF-α molecular mechanism in normoxia (**a**) and hypoxia (**b**). Under normoxic conditions the synthesized cytoplasmic HIF-α is hydroxylated by prolyl hydroxylase (PHD) enzymes on proline residues 402 and 564 located within the oxygen-dependent degradation (ODD) domain and by the Factor Inhibiting HIF-1 (FIH-1) on asparagine. This hydroxylation is the key mechanism of HIF-α-negative regulation activity since it determines its binding with the von Hippel-Lindau (VHL) E3 ligase complex, which labels HIF-α with ubiquitin allowing its recognition by the proteasome and therefore its degradation. In this way the transcription of hypoxia gene target cannot proceed. **b** Under hypoxic conditions, the availability of substrates, coactivators of hydroxylation and O_2_, becomes limited and their deficiency determines the attenuation of HIF-α hydroxylation by enzymes as PHD and FIH. HIF-α can accumulate in the cytosol and subsequently translocate to the nucleus where it dimerizes with the HIF-β subunit. The HIF-α/β dimer is able to bind to Hypoxia-Response Element (HRE) and it allows to initiate the transcription of genes target as EPO, IGF-2, TGF-α, VEGF, MMP2, αβ integrins, and genes involved in glucose metabolism. Retrieved from https://app.biorender.com/biorender-templates (2022)

The cells respond to oxygen level changes through the activation of genes involved in angiogenesis, glucose metabolism, and cell proliferation/survival processes. The molecular protagonist of eukaryotic cellular response is hypoxia-inducible factor-1α (HIF-1α), which acts as a primary transcriptional mediator by regulating oxygen homeostasis [87].The α subunit of HIF-1 (HIF-1α) was originally identified as a nuclear factor that binds to the 3′ enhancer region of the erythropoietin (EPO) gene and regulates its expression in response to low levels of blood oxygen [135]. Three isoforms of HIF-α (HIF-1α, HIF-2α, and HIF-3α) and two β subunit isoforms of HIF (ARNT or HIF-1β and ARNT2) have been discovered so far. All of them have a similar domain structure and belong to the bHLH-PAS protein family. The motifs PAS and bHLH are required for heterodimerization between the HIF-1α and β subunits, which is necessary for them entering into the nucleus and inducing the expression of multiple genes [22]. The transcriptional activity of HIF-1α followed by its interaction with coactivators such as CBP/p300 are mediated by C-TAD and N-TAD domains present in the C-terminal region [51, 104]. Moreover, HIF-1β is a constitutively expressed subunit, while HIF-1α, after translation, is degraded via ubiquitination-driven proteasomal process under normoxic conditions [49, 106]. On the other hand, HIF-α is stabilized under hypoxic conditions. In detail, during normoxia the synthesized cytoplasmic HIF-1α is hydroxylated by prolyl hydroxylase (PHD) enzymes in proline residues 402 and 564 located within the oxygen-dependent degradation (ODD) domain [52, 121]. This hydroxylation is the key mechanism of HIF-1α-negative regulation activity since it determines its binding with the von Hippel-Lindau (VHL) E3 ligase complex, which then labels HIF-1α with ubiquitin and allows its recognition by the proteasome which eventually leads to its degradation [77, 78, 81]. Another important mechanism that modulates the HIF-1α activity is the asparagine 803 residue (Asn803) hydroxylation in the C-terminal transcriptional activation domain (C-TAD). Asparagine is hydroxylated under normoxic conditions by the Factor Inhibiting HIF-1 (FIH-1), which does not permit the interaction of HIF-1α with CBP/p300 (CREB binding protein/E1A p300 binding protein) [65, 66, 74] (Fig. 1). In hypoxic conditions, the availability of substrates, coactivators of hydroxylation and oxygen, become limited and their deficiency determines the attenuation of HIF-1α hydroxylation [53, 107]. HIF-1α can accumulate in the cytosol and subsequently translocate into the nucleus where it dimerizes with the HIF-1β subunit. The HIF-1α/β dimer is able to bind with Hypoxia-Response Element (HRE) through functionally essential HIF-1-binding sites of the consensus sequence 5′-RCGTG-3′ in the sequences of oxygen-regulated genes and influences their expression [19, 50, 100].

Several HIF-1 target genes encoding proteins are involved in different cellular processes ranging from stress response to angiogenesis mechanisms that allow cell/tissue adaptation to hypoxia. For example, in tissue hypoxia conditions HIF-1 upregulates the expression of EPO which is necessary for red blood cell production, induces an increase in erythrocyte number followed by the oxygenation of the tissues [115]. Furthermore, HIF-1 influences gene expression involved in glucose metabolism as the cells under hypoxic can produce ATP exclusively through the glycolysis and generates only two molecules at the expense of the 38 obtained from the oxygen-dependent tricarboxylic acid cycle under normoxic condition [110, 146]. Since the energy balance has to be maintained precisely even in hypoxic condition, HIF-1 increases the expression of glycolytic enzymes and glucose transporters to allow the cells to increase glucose absorption and to obtain the required ATP [113, 116, 139]. In addition, cell proliferation and survival factors are upregulated by HIF-1, such as insulin growth factor-2 (IGF-2) and transforming growth factor-α (TGF-α) [27, 64]. Moreover, current studies have reported that hypoxia and HIF-1 expression influence the angiogenesis process in which HIF-1 transcriptionally activates vascular endothelial growth factor A (VEGF-A) and other angiogenic genes with related receptors, inducing the development of new vessels [39, 111]. In addition, to facilitate the endothelial progenitor cells recruitment to the hypoxia site HIF-1α enhances the expression of metalloproteinase 2 (MMP2) to trigger the extracellular matrix (ECM) degradation and to allow endothelial cell migration [12]. Subsequently, HIF induces the expression of αβ integrins that stimulate the endothelial cell proliferation and adhesion [55].

## Tissue hypoxia: pathological consequences

An alteration in blood flow results in a lower oxygen flow to the organ. This condition can cause damage to the cell and the subcellular organelles, resulting in cellular damage and that the damage is reversible if the hypoxic stimulus is short-lived; otherwise, if the damage persists for longer period the biochemical balances are altered, which leads to cell death followed by the destruction of the tissue via various pathological consequences depending on the affected organ [120].

### Hypoxia in cardiac disease

One of the tissues that are most affected by the changes in oxygen levels and by hypoxic state is certainly the heart.

This is mainly due to the fact that oxygen contributes to nitric oxide (NO) formation by mediating cardiac and vascular contractility through the oxygen-dependent S-nitrosylation process of cysteine residues [9]. This results in the excitation–contraction coupling and the control of calcium flow, and thus influences homeostasis, contractility, and response to injury [8, 92]. Moreover, oxygen is involved in reactive oxygen species (ROS) formation, which on one side is a positive event for the establishment of certain processes such as differentiation into myofibroblasts, and on the other side, it represents a negative event since it promotes the cell death through mitochondrial damage [123, 128]. In addition, oxygen is used in many enzymes’ activity, especially Cytochrome Oxidases such as P450 [131]. The increase in Nicotinamide Adenine Dinucleotide Phosphate (NADPH) oxidase activity can contribute to the formation of atherosclerosis because it generates superoxides which lead to low-density lipoprotein oxidation with consequent endothelial dysfunction and foam cell formation [5]. Taken altogether, HIF impacts a number of cardiac phenotypes; indeed, many events that occur as a result of HIF stabilization and accumulation are cardioprotective and are believed to be an adaptive ischemic preconditioning (IPC) mechanism to hypoxia. This latter event, through short-period exposure of non-lethal hypoxia alternated with myocardial reperfusions, would appear to protect the heart from major myocardial damage [85]. Conversely, chronic exposure to hypoxia could be harmful to heart tissue, compromising calcium handling and thus cardiac metabolism. This can lead to heart failure [43].

### Hypoxia in cancer

In the major part of the tumors an overexpression of HIF-1α protein [114] was found, highlighting how a limited oxygenation in the tumor microenvironment supports the development of the tumor itself and specifically a passage towards metastatic form. In particular, in some solid tumors (bladder, brain, breast, colon, esophagus, head and neck, liver, lung, pancreas, skin, stomach, and uterus cancers) and hematological (acute lymphocytic and myeloid leukemias), HIF overexpression is associated with a poor prognosis [112]. Hypoxia through HIF is a strong regulator of ubiquitination process whose proteasomal degradation regulates protein homeostasis. The tumor growth is supported by an alteration of the degradation processes that determine a greater or lesser synthesis of specific proteins [82]. In detail, as the tumor mass grows (solid tumors) the oxygen disposition for the cells, especially in the mass internal part, decreases. This determines “the Warburg effect,” the phenomenon by which the neoplastic cells change their metabolism to produce adenosine 5′-triphosphate (ATP) using exclusively the anaerobic glycolysis [130], unlike normal differentiated cells which rely mainly on mitochondrial oxidative phosphorylation to generate the energy needed for cellular processes. Initially, Warburg had hypothesized that tumor cells exploited anaerobic respiration because they had defects at the mitochondrial level [137], but since these alterations are not found in all tumor types, it is possible that at the basis of this metabolic switch could be other reasons [68, 138]. A possible explanation is tumor hypoxia, as stated, which selects cells dependent on anaerobic metabolism. However, this explanation is not sufficient for all cancer types, because some of these make the metabolic switch before the onset of hypoxic condition. Furthermore, hematological tumors, particularly leukemias, are characterized by the cells with an enhanced glycolytic rate despite residing in the bloodstream with high oxygen tensions [26]. Tumor hypoxia is fundamental not only for metabolic changes in cancer cells but also for the metastasis insurgence [83]. Previous in vitro studies reported that hypoxia is associated with an expression and activity increase of type IV collagen-degrading enzymes MMP2 and MMP9 [34]. These metalloproteinases are upregulated by hypoxia in breast and colon cancer cells via HIF1-dependent mechanism, while type 1 MMP, also known as MMP14, exhibits HIF2-dependent upregulation [34]. This indicates that hypoxic cancer cells facilitate tumor progression by degrading the matrix collagen through MMPs. In addition, neoplastic cells show a greater proteolytic activity via increased expression of urokinase plasminogen activator surface receptor (PLAUR) in a HIF-dependent manner [16]. PLAUR promotes cell invasion by altering the interaction between integrins and ECM.

When shRNAs regulate PLAUR expression, the cells are incapable of intravasation [58]. Simultaneously with basal degradation the HIFs activate a transcriptional program which also determines the increased ex novo fibrillar collagen synthesis to constitute binaries for tumor invasion [17]. As HIF-1 is a transcriptional activator of VEGF-A and other angiogenic genes, hypoxia has influence on tumor angiogenesis. Furthermore, experimental evidence indicates that the same molecules and growth factors involved in the angiogenesis processes driven by hypoxia also determine fibroblast or myofibroblast recruitment in the pathological site [13]. VEGF-A released by hypoxic cancer cells is implicated in fibrosis, suggesting the role of hypoxia in the activation of stromal cells and in the production of ECM. By regulating microvascular permeability, hypoxia-driven VEGF-A consequently influences the influx of fibroblasts, inflammatory cells, and endothelial cells to the primary tumor, supporting tumor growth [15]. The vascular stroma generation is therefore an essential feature of solid tumor growth and is correlated with a better or worse prognosis depending on the microvascular density [28].

### Hypoxia in brain damage

Based on the literature hypoxia may play a role in multiple sclerosis (MS) pathogenesis [37]. Previous studies reported that HIF-1α expression was found in multiple sclerosis and in white matter stroke (WMS) [2, 122]. Furthermore, hypoxia can be a critical event in the context of MS since the signaling pathways for hypoxia and inflammation are interconnected by the enzyme prolylhydroxylase (PHD), which is responsible for breaking down HIF-1α [10]. The PHD pathway is correlated with the NF-κB, because both PHD and FIH jointly regulate NF-κB activation by controlling the activity of the beta subunit of the IκB kinase complex (IKKβ) that phosphorylates the nuclear factor-kappa B inhibitor alpha (IκBα), which eventually liberates NF-κB for translocation into the nucleus where it can activate the transcription of inflammatory genes and proinflammatory cytokine production [10]. Thus, hypoxia allows to stabilize both HIF-1α and NF-κB, leading to upregulation of genes involved in the hypoxic and inflammation mechanism, causing vascular damage and favoring the leukocyte influx [62]. This mechanism is generated by the inflammatory cell state, which leads to a greater metabolic demand and increase the oxygen demand, exacerbating the hypoxic state. In MS patients, a reduced vasodilation and decreased cerebral blood flow was found to be significantly correlated with gray matter (GM) loss and cognitive impairment, suggesting that an insufficient blood flow, which induces a hypoxic stress, may play an important role in neurodegeneration. Thus, hypoxia/ischemia-induced metabolic stress leads to impair neuronal function [47, 76]. This state is supported by the mitochondrial alterations caused by the hypoxic state. Indeed, the structural and functional mitochondrial damage has been found in acute lesions of MS [73]. Moreover, it is important to mention that in the established inactive MS plaques, the total number of mitochondria and their activity are more evident, confirming the increase in energy demand in stressed and demyelinated axons [141].

### Hypoxia in inflammatory bowel disease

Hypoxia plays a decisive part in inflammatory bowel disease (IBD) due to the key role played by HIF-1 in promoting an inflammatory microenvironment in myeloid cells in Crohn’s disease (CD) and ulcerative colitis (UC) [6]. CD has unknown etiology associated with an impaired immune response. It is characterized by periods of activity and remission and shows patchy and transmural lesions which can affect the entire gastrointestinal tract [7]. UC is another chronic inflammatory bowel disease that affects the colon and rectum and like CD its etiology is unknown. It has been suggested that multiple factors (genetic background, environmental and luminal factors, and immune dysregulation of the mucosa) contribute to the pathogenesis of UC [61]. Hypoxia is a feature of the inflammatory condition especially due to the oxidative burst of the neutrophils. This microenvironment has the ability to activate hypoxia-dependent transcriptional responses within distinct cell subtypes, which shape the inflammatory locus through alteration of cell metabolism, cytokine release, subtype differentiation, and cell survival [127].

Recently, claudin-1 (CLDN1), a fundamental molecule in the tight junction formation on the apical surface of epithelial cell, has been identified as a HIF target, capable of rescuing the typical compromised intestinal barrier of IBD [105]. It is well known that creatine kinase enzymes, located in adherens junctions, are expressed in intestinal epithelial cells in a HIF-2 α-dependent manner [35]. Contributing to barrier protection there are also other components such as mucin MUC-3 and intestinal trefoil factor, which appear to be HIF dependent [31, 71]. Human β-defensin 1 (hBD1), an anti-microbial peptide that contributes to protective effect, is constitutively expressed by epithelial cells in HIF-dependent manner and its activity is promoted by hypoxia conditions [56].

On a clinical point of view HIF-1α and HIF-2α levels are elevated in the intestinal epithelial cells of patients with CD and UC [145]. In particular, in biopsies of patients with UC in remission and with active disease, a correlation between the expression of HIF and the severity of the disease was found [144].

### Hypoxia in lung disease

Normally hypoxia affects lung tissue at the fetal level in certain conditions. Fetal oxygen supply may be reduced due to decreased uterine blood flow (such as in the case of placental insufficiency or a smoking mother) or due to decreased oxygen supply of the mother (such as at high altitudes). This hypoxia state in the fetus leads to pulmonary hypertension with increased airway resistance and an inhibition of fetal respiratory movements [42]. Previous studies reported that babies of mothers who smoke have had abnormally small lungs and their function is significantly impaired for the first 18 months of life [44]. Furthermore, in children who died of sudden infant death syndrome (SIDS) and whose mothers smoked more than 20 cigarettes per day, there was an increase in the thickness of the airway walls and a decrease in lumen [24].

An alteration of the airway development and of the vasculature at postnatal level occurs above all in those premature infants who have been subjected to mechanical ventilation. The latter seems to compromise pulmonary development, also confirmed by the histological aspect that reflects the organ immaturity degree at birth and the response to long-term ventilatory support [18]. The peripheral arterial and alveolar development is interdependent both in normoxic and hypoxic conditions. Experimentally the addition of a VEGF-A receptor inhibitor or of anti-angiogenic factors determines a reduction in the arterial and alveolar system development in growing animal models [48], which is confirmed by the fact that newborns with bronchopulmonary dysplasia (BPD) appear to have a reduction in VEFG and its receptor [75]. In adults the hypoxia state induces a response of pulmonary endothelium which actively participates in such condition by integrating sensory and effector functions that allow to modify the blood flow and vascular pressure. In particular, hypoxia can induce endothelium lesions or dysfunctions, causing an increase in the vascular smooth muscle permeability and tone, and cell proliferation, promoting a thrombotic state [129]. Acute hypoxia induces reversible constriction of vascular pulmonary smooth muscle cells (PASMC) by inhibiting voltage-gated K + (Kv) channel activity in PASMC [96]. Specifically, hypoxia triggers a biphasic pressure response, an early one followed by vasorelaxation, and a second wave of persistent contraction; this second stage can represent the true basis of hypoxic pulmonary vasoconstriction (HPV) [33]. Chronic hypoxia causes pulmonary hypertension (PH), which consists of an increase in mean pulmonary arterial pressure to over 25 mm Hg at rest or over 30 mm Hg during exercise or cardiac catheterization [54, 91]. PH, associated with several lung diseases (sleep apnea and chronic obstructive pulmonary disease (COPD)), has been associated to chronic hypoxia [129]. Lung endothelial cell dysfunction may represent evidence that pulmonary vascular remodeling in severe PH involves elements of abnormal angiogenesis similarly as observed in neoplastic cells, where HIF-1α is the key molecule in the angiogenesis control and tumor growth [132, 150]. Previous works reported that in hypoxic pulmonary vascular cells, mitochondrial ROS production decreases, inhibiting oxygen-sensitive channels and thus causing membrane depolarization. This leads to the voltage-gated calcium channel activation and calcium influx, initiating HPV [103]. If this redox oxygen sensor is damaged, the affected mitochondria create a false “hypoxic signal” which leads to chronic activation of HIF-1α even in normoxia. At this point HIF downregulates the oxygen-sensitive channels, causing the trigger of a cascade activation which leads to PH. This mechanism may not only lead to an increased vasoconstriction, but also lead to apoptosis resistance with hyperpolarization of the mitochondria [80], creating a connecting bridge between different pathological conditions.

### Hypoxia in kidney disease

Although kidney is one of most perfused organs in the body in relation to its weight, the oxygenation of the renal parenchyma is poor (for example, in the rats the oxygen tension of the renal cortex is 30 mm Hg, and in renal medulla it is less than 10 mm Hg [72, 109]). The reason of this state is related to the regional blood flow to the renal medulla which is lower than the renal cortex. Furthermore, the architecture of the renal vessels influences this paradox since the preglomerular and postglomerular arterial and venous vessels run parallel and in close contact for long distances, guiding the diffusion of oxygen from arterioles to the postcapillary venous system before it can enter the capillary bed [151]. The renal tubules are characterized by their limited capacity to generate energy in anaerobic conditions and rapid consumption of oxygen in the metabolic processes [30]. Therefore, renal architecture and high oxygen demand combined to make the kidney particularly susceptible to the conditions that cause ischemia. This state, in addition to problems of anemia, hypertension, and kidney damage, can determine the hypoxia in the kidney [85].

In particular, one of the major causes of renal hypoxia is acute kidney injury (AKI) in which the hypoxic state is established not only during the acute phase of AKI, but also after the recovery phase as a prolonged hypoxia that results in the downregulation of the proangiogenic isoform 164 and the dysangiogenic isoforms 120 and 188 which upregulate VEGF-A [11]. Consequently, failure of the vascular architecture of the kidney occurs with a decreased number and the caliber of capillaries [79]. In detail, the initial hypoxic insult from AKI episode consolidates with the alteration of the vascular system and induces chronic hypoxia, which apparently becomes the cause of a series of pathological processes affecting tubular epithelial cells and inducing apoptosis. Moreover, hypoxia causes changes in gene expression by inducing leukocyte β2 integrin expression and function by transcriptional mechanisms dependent upon HIF-1 that has a binding site in the CD18 gene. This gene encodes the subunit common to all four known types of β2 integrin heterodimer. HIF-1 binding with the CD18 promoter results in a loss of hypoxia inducibility [63, 148]. This expression is mediated not only by chromatin remodeling and histone modification but also by the transcription factor HIF-1, resulting in hypoxia-dependent gene regulation [63].

Hypoxia caused macrophage accumulation in the renal tissue which produced profibrotic cytokines by activating renal fibroblasts, which are also activated directly by hypoxia to increase the deposition of ECM [89]. Fibroblast activation, together with inflammatory cell recruitment and tubular epithelial cell damage, leads to tubule-interstitial fibrosis. The latter aggravates the hypoxic state, which eventually leads to chronic kidney disease (CKD). Moreover, the tubule-interstitial fibrosis is aggravated by the augmentation of endothelin expression then vasoconstriction [126, 149].

### Hypoxia in liver disease

The acute and chronic liver disease etiologies are diverse and include hepatitis infection, portal hypertension, and fatty liver disease, which are developed mainly in association with obesity or as a result of alcohol abuse. This condition can lead to important pathologies such as steatosis and steatohepatitis, which can evolve into hepatic fibrosis, cirrhosis, and hepatocarcinoma. Hypoxia appears to exacerbate the non-alcoholic fatty liver disease through the overexpression of HIF-2α which alters lipid metabolism [20]. In addition, a new HIF1α/PTEN/NF-κBp65 signaling pathway has been identified recently in non-alcoholic fatty liver disease in which HIF-1α promoted fibrosis via PTEN/ p65. This could represent a promising therapeutic target [41]. In liver diseases the angiogenesis process plays a key role, which has a double effect: in the initial repair and revascularization phase of the damaged parenchyma, angiogenesis has a positive effect, but in the subsequent phase there is a localization of the expression of cytokines from endothelial cells only in the points where they stabilize the newly formed vessels. This results in an enlargement of the vascular tree, which cause an increase in blood flow and thus cause and/or exacerbate portal hypertension [25]. On one hand, hypoxia seems to aggravate the chronic liver damage, and on the other hand, HIF-2α reprograms the hepatic macrophages to protect them from acute liver injury [32]. Interestingly, short periods of ischemia may be able to protect liver tissue from subsequent damage, especially to cope with liver ischemia–reperfusion (I/R) injury in both liver resection surgery and liver transplantation. The hypoxia/reperfusion alternation is called ischemic preconditioning and appears to reduce tissue necrosis and apoptosis, proving to be a protective and survival mechanism [117].

## Hypoxia in tissue repair and regeneration

Hypoxia is involved in various processes, especially in the angiogenesis mechanism. Also, it plays a fundamental role in tissue repair and regeneration [45]. Acute hypoxia has been found to stimulate wound healing; however, the recovery of normal tissue oxygenation is necessary to avoid a chronic hypoxia state which instead compromises the healing process [108]. In detail, during the inflammatory phase, oxygen is essential for the various processes that require ATP through oxidative phosphorylation in the mitochondria. After hemostasis, hypoxia activates the early stages of wound healing through HIF-mediated regulation [59]. Furthermore hypoxia causes increased ROS activity which eventually lead to the activation of platelets and monocytes to release cytokines and growth factors transforming growth factor beta one (TGF-β), VEGF-A, and tumor necrosis factor-α (TNF-α) [36]. The main consequence of these mediators is the neutrophil and macrophage recruitment into the wound site and subsequent fibroblast activation [36, 40]. TGF-β1 is responsible for the transcription of the procollagen gene, which is crucial in the tissue repair process because it increases the migration of fibroblasts [84]. Acute hypoxia has been shown to increase TGF-β1 messenger RNA expression, followed by collagen synthesis and fibroblast proliferation [119]. However, oxygen is needed in the posttranslational steps of collagen synthesis which are directly dependent on O_2_ as they are mediated by enzymes that require the molecular cofactor O_2_. Prolyl hydroxylase is an enzyme that converts proline residues into hydroxyproline, allowing the procollagen peptide chains to attain their triple-helix configuration. In the absence of this triple-helix configuration, synthesized procollagen chains accumulate in the rough endoplasmic reticulum and are finally excreted as a non-functional gelatinous protein [99]. Once the procollagen has attained the triple-helix conformation and excreted, the single-collagen fibers are arranged in linear fibrils via cross-linking by lysyl hydroxylase and finally the cross-linking between the large fibrils occurs by lysyl oxidase, which are the other two oxygen-dependent enzymes involved [29]. This is one of the reasons why it is necessary to restore normoxia following an acute state of hypoxia as the chronic state compromises the angiogenesis of the wound and causes death and tissue dysfunction [60]. Another reason is that although hypoxia stimulates angiogenesis by inducing VEGF-A expression, the process is sustained over time only when VEGF-A is released at higher oxygen voltages [94, 118].

Recent studies on experimental models focused on the effect of hypoxia in tissue regeneration and repair. Yuji Nakada et al. reported that mice exposed to a state of systemic hypoxia inhibited the oxidative metabolism, decreased the production of ROS and oxidative damage to DNA, and reactivated the mitosis of cardiomyocytes. In particular, exposure to hypoxia for 1 week after induction of myocardial infarction induced a decrease in myocardial fibrosis and an improvement in left ventricular systolic function with a robust regenerative response. Genetic fate-mapping analysis confirmed that the newly formed myocardial tissue was derived from the preexisting cardiomyocytes. These data demonstrated that exposure to gradual systemic hypoxemia was able to reactivate the endogenous regenerative properties of the adult mammalian heart [86].

Hypoxia seems to have an important role in neuronal regeneration. It has been reported that HIF-1α induction by hypoxia improved the regeneration of axons in periferic neurons, while HIF1A knockdown in in vitro or conditional knockout in in vivo models exhibited the compromised sensory axon regeneration. VEGF-A, a target gene of HIF-1α, has been found to be expressed in damaged neurons and helps to stimulate the regeneration of the axons also in the motor neurons, accelerating the reinnervation of the neuromuscular junction. These data suggest that HIF-1α represents a critical transcriptional regulator for both neuron and axon regeneration [21].

Neuroprotective effect of hypoxia has been investigated in the preclinical models. In a rat model, the preconditioning of intermittent hypobaric hypoxia (IHHP) not only improved the regeneration and repair capacity but also attenuated the neurological deficit as well induced by the reperfusion of cerebral ischemia. These results suggest that IHHP can be useful in the clinical setting approach [143].

Similarly, a study in rat models showed that drug-induced hypoxia accelerated the liver regeneration in a similar way to that achieved by portal vein ligation and parenchymal section. Therefore, the hypoxia process also seems to be able to accelerate the regeneration of hepatic tissue, which is one of the tissues with the highest degree of regeneration [38]. Moreover, hypoxia has been reported to act as a hepatoprotector, protecting liver from acute liver damage. This hepatoprotective effect has been shown to occur through HIF-2α-mediated reprogramming of liver macrophages [32]. Furthermore, hypoxic stimulation in hepatic stellate cells (HSC) lead to an induction of hepatic sinusoidal endothelial cell proliferation (LSEC) in vitro guided by VEGF-A. In vivo, portal vein ligated plus transection rat models revealed that rapid regeneration was also associated with an increase in von Willebrand factor [23].

### Hypoxia and biomaterials

Hypoxia appears also to be a promising process for tissue regeneration through biomaterials used in tissue engineering. For example, Yaling Yu et al. reported the effect of hypoxia on renal tissue regeneration mediated by decellularized renal scaffolds (DC).This study demonstrated that HIF-1α is a major contributor to DC scaffold-mediated renal regeneration; in detail, after the implantation of the scaffold in the damaged kidney of animal models the glomeruli were induced to regenerate with an upregulation of HIF-1α which lead to the recovery of renal function. This was confirmed by the fact that in rats DC scaffolds treated with HIF-1α siRNA showed reduction in renal regenerative capacity due to the silencing of HIF-1α expression. To verify these effects, a counter-test was made with a treatment that stabilized HIF-1α expression with the consequent increase in renal regeneration. These findings showed HIF-1α as a key molecule in mammalian renal regeneration induced by the DC scaffold [147].

Previous studies have focused the attention on hypoxia as a possible tool for improving the usage of the scaffolds in osteochondral tissue engineering, inducing the regeneration of damaged cartilage and its subchondral bone. The scientific community has been orientated in the development of a biomaterial that is able to spatially control the stabilization of HIF-1α cofactor, to stimulate a region-specific articular cartilage formation in which HIF-1α is active, and to promote the formation of the subchondral bone region in which HIF-1α activity is blocked, within a single construct. This construct may mimic physiological condition of the native cartilage which is under low-oxygen conditions and the subchondral bone which is relatively normoxic [124].

Another in vitro and in vivo study analyzed the effect of hypoxia preconditioning of adipose-derived stem cells (ADSC) in the reconstruction of the urethra using new scaffolds. The data showed that hypoxia-preconditioned ADSCs combined with the new porous nanofiber scaffold resulted in an increase in the diameter of the urethral lumen, which is a preservation parameter of the urethral morphology, and an improved angiogenesis compared to the cells combined with the same scaffold but preconditioned in normoxia. Thus, hypoxia preconditioning of ADSCs combined with scaffold promotes urethral reconstruction by upregulating angiogenesis and glycolysis. This may be a promising alternative treatment for urethral damage [133].

### Hypoxia and stem cells

Another fundamental aspect of tissue regeneration is the application of stem cells. Precisely for this reason it is also advisable to analyze how mesenchymal stem cells (MSCs) react to the hypoxic state. Since hypoxia is a condition of low oxygen levels the first cellular consequence will be at metabolic level. It was found that in MSCs, after the exposure to hypoxia, there was a significant upregulation of the glycolytic proteins with an increased absorption capacity of glucose through glucose transporter GLUT1 (*SLC2A1*) and fructose transporter (GLUT5/*SLC2A5*). Conversely, mitochondrial proteins such as citric acid cycle proteins (TCAs) and enzymes involved in the degradation and transport of fats and amino acids and in the transport chain of electrons (ETC) were downregulated. Mitochondrial ribosomal proteins (MRP family) were also decreased. Furthermore, hypoxia influences glycogen turnover through the glycogen synthase and glycogen phosphorylase L upregulation [142]. Interestingly, several studies evidenced that hypoxia increased the regenerative properties of MSCs in different tissues [1, 3]. At the neuronal level, bone marrow-derived mesenchymal stem cells (BMSCs) exposed to hypoxic conditions showed increased differentiation potential. In particular, in vitro tests revealed that hypoxic BMSC culture induced an increase in the expression of RNA and proteins of neuronal markers. Electrophysiology study also showed better results in BMSC-differentiated neuronal cells cultured under hypoxia than in normoxic control. This was further corroborated in in vivo models which showed that the potential treatment of rat hypoxic BMSCs (rBMSC) led to better results in regeneration than in normoxic rBMSCs. This study reported that myelinated nerve fibers were present more in numbers in the hypoxic rBMSC group than in the normoxic group. Accordingly, it can be stated that BMSCs grown in hypoxia may have a greater potential for neuronal differentiation both in vivo and in vitro. Indeed, this is a fundamental requirement in a therapeutic perspective for repairing peripheral nerves, which is a challenging undertaking till date due to some disadvantages of nerve grafts [136]. With regard to skeletal muscle regeneration many lines of evidence showed that oxygen concentration regulated the proliferation and differentiation of satellite cells which are responsible for skeletal muscle reconstruction. In particular, BMSC cultures, under hypoxic conditions, increased the VEGF-A expression and myogenesis. Transplantation of hypoxia-preconditioned BMSCs into the damaged muscles in in vivo models improved the cell engraftment performance via the formation of new vessels. Among the molecules fundamental for better engraftment and angiogenesis, SDF-1 and VEGF-A secreted by the MSCs in question have been identified [4]. Fang Wang et al. reported that 5% hypoxia exposure increased the differentiation of adipose tissue-derived MSCs into smooth muscle cells. This differentiation was supported with the increased expression of α-actin, calponin, and myosin heavy chain. Moreover, enhanced contractile capacity was noticed. These data suggest that adipose tissue is a valuable resource for regeneration of gastrointestinal tissues, such as intestine and sphincters [134]. In general, the low-oxygen environment modulates the differentiation towards endothelial cells (EC). This has been demonstrated by in vitro studies in which low-oxygen-conditioned pluripotent stem cells obtained from different sources showed differentiation towards endothelial lineage as evidenced by the formation of typical endothelial-like tubulo-structures and the expression of typical endothelial markers. These results were also validated by in vivo studies with mouse and rat models treated with injections of endothelial differentiated stem cells that improved the blood perfusion in the ischemic hind limb and cardiac function after myocardial infarction (MI) [97]. Interestingly, hypoxia pretreatment significantly increased chondrogenic differentiation from the MSCs, but decreased osteogenic differentiation compared to normoxic controls. This could also reflect the physiological condition of these tissues. Furthermore, the addition of VEGF-A to the differentiating ADSC cultures decreased the osteogenic differentiation which, on the other hand, was increased with VEGF-A depletion [46]. Recently, it was found that HIF-1α overexpression promotes renal stem cell proliferation, while the HIF-1α inhibition stops this proliferation. Furthermore, the overexpression of HIF-1α not only induces the proliferation but also leads to a reduction in renal stem cell apoptosis, while reduced HIF-1α increases the apoptosis of these cells. Moreover, a positive correlation was identified between renal stem cell marker CD133 expression and HIF-1α expression: an overexpression of HIF-1α increases CD133 expression, while reduced HIF-1α expression decreases expression of CD133 [69]. Several studies showed that hypoxia promotes different responses in MSCs derived from various sources.

Earlier studies evaluated the hypoxia effects on the proliferation, apoptosis, and expression of genes related to the pluripotency of stem cells of human exfoliated deciduous teeth (SHED). The results showed greater expression of pluripotent genes after 24 h and 7 days in cells that were cultured under hypoxia condition compared to those grown in normoxia. On the other hand, no significant differences were found between cell cultures from human exfoliated deciduous teeth under hypoxia and normoxia with regard to metabolic activity, proliferation rate, and apoptosis. An increase in the pluripotency of the SHED culture in hypoxia has been indicated as a major advantage [140].

Although on one hand the positive effect of hypoxia on MSCs of different derivation has been identified, on the other hand hypoxia supports neoplastic stem cells in some types of tumors, showing a protumoral effect. For example, in glioblastoma angiogenesis and therapeutic resistance are modulated by hypoxia as well as tumor microenvironment. In glial tumors, growth, angiogenesis, and therapeutic resistance itself are supported by glioma stem cells in which hypoxia upregulated the expression of stemness markers (CD133, OCT4, SOX2) and promoted CD133 glycosylation, which may play a role in the hypoxia-mediated anti-apoptosis process. It is imperative to mention that HIF1α stabilization leads to the glioma stem cell (GSC) population expansion within the tumor mass through Phosphatidyl Inositol 3-Kinase (PI3K)/Protein kinase B (PKB or AKT) pathway. The stemness maintenance appears to be mediated by HIF1α through signal transducer and activator of transcription 3 (STAT3) and Notch1 stabilization as well as by VEGF-A transcription. HIF1α promotes GSC self-renewal and represses differentiation to increase glioblastoma growth [14]. Hypoxia appears to be important not only for maintaining GSC, but also for guiding the process of metastasis. Accordingly, inhibition of the hypoxic pathway could represent a strategy to prevent invasion of the brain by GSC [93]. Indeed, pathogenic role of HIF-1α was observed in the specimen leukemic stem cells/lymphomas. In particular, a correlation was identified between HIF-1α expression, therapeutic outcome, and prognosis. Recent studies reported that in mouse lymphoma model and human acute myeloid leukemia (AML) stem cells aberrant activation of HIF-1α was observed, which led to the stimulation of its signaling cascade even under normoxic conditions. Inhibition of HIF-1α or overexpression of VHL leads to the colony formation inability in the same models, in which the presumed fraction of lymphoma stem cells activates HIF-1α while also increasing Notch expression, suggesting that HIF-1α may represent a potential target for hematological malignancy treatment [125]. In colorectal cancer (CRC) cells, hypoxia has been shown to increase the self-renewal capacity of tumor-initiating cells while inducing the arrest of the proliferation of more differentiated cells. Thanks to gene expression analysis, hypoxia has been found to induce autophagy in the tumor initiator cells, which mediates their self-renewal via Protein kinase C (PKC) and EZR phosphorylation. The hypoxia-autophagy-PKC-EZR signaling axis represents a new regulatory mechanism of self-renewal for the onset and progression of colorectal cancer [57]. In addition, CD133-expressing colorectal cancer cells exhibited increased HIF-1α expression and accentuated tumor cell migration during hypoxia compared to CD133-negative cells. This has been associated with a greater epithelium-mesenchymal transition capacity, which predisposes to an increase in the hematogenous metastatic potential and thus the formation of hepatic metastases. It appears that in hypoxic CD133 − cells, integrin β1 expression levels play a key role in cell adhesion to the peritoneum, resulting in metastasis at this level [102].

Hypoxia may also affect the efficacy of some therapeutic agents such as histone deacetylase (HDAC) inhibitors in breast cancer. Specifically, hypoxia has been shown to preferentially block the differentiation induced by the HDAC inhibitor of the BRCA1-reconstituted breast cancer cells. BRCA1-reconstituted tumor cells have been found to be more sensitive to HDAC inhibitor-induced stem loss than BRCA1-deficient cells. Accordingly, BRCA1 status and tumor hypoxia should be considered as key clinical parameters which may affect the therapeutic efficacy of the HDAC inhibitors [90]. MSCs under hypoxic conditions also play a key role in the hepatocellular carcinoma (HCC) progression. Hypoxia has been shown to support the progression of this neoplasm through the Cyclooxygenase 2 (COX2)/Prostaglandin E2 (PGE2)/prostaglandin E receptor 4 (EP4)/yes-associated protein (YAP) axis. In turn, YAP in HCC cells activates the AKT/Mechanistic target of rapamycin (mTOR)/sterol regulatory element-binding protein 1 (SREBP1) pathway which accelerates HCC cell growth by lipogenesis induction [70]. Another neoplastic condition in which hypoxia appears to support tumor stemness and drug resistance is non-small-cell lung carcinoma, in which the hypoxic state contributes to confer cisplatin resistance by overexpressing Tie1 in a HIF-1α-dependent way [67]. This mechanism of crosstalk between hypoxic tumor environment and neoplastic stem cells could also serve as a target for the new treatments.

## Conclusions

Through the elucidation of multiple current studies, the complex roles of hypoxia and its involvement in multiple conditions have been highlighted. It is evident that the hypoxic state can be a positive and potentially therapeutic process in various pathologies but at the same time it is also characterized by its pathological contribution that ranges from neurodegenerative conditions to neoplasms. The current review was aimed at analyzing the molecular mechanisms and gene targets of hypoxia as an aggravating factor in various pathological contexts with important consequences. Moreover, our review has explained how hypoxia can be identified as a positive process in tissue repair and regeneration with respect to the cellular stemness property. On one hand, the involvement of hypoxia on the stemness maintenance it is advantageous for regeneration process and on the other hand, it can be harmful when it is concerned about the neoplastic stemness maintenance, which determines the greater tumor aggressiveness and metastasis capacity. Further studies are needed to clarify many unresolved complex molecular processes that allow the exploitation of hypoxia as a tissue repair and regenerative tool and as a target for new molecular therapies.
